# The H5N6 Virus Containing Internal Genes From H9N2 Exhibits Enhanced Pathogenicity and Transmissibility

**DOI:** 10.1155/tbed/6252849

**Published:** 2025-01-06

**Authors:** Manlin He, Lina Liu, Jinglei Hu, Zhenjun Wang, Zhendong Guo, Xiaohan Wang, Yongyang Sun, Shaowen Shi, Wenhao Ren, Yuxing Wang, Xiaoxuan Nie, Chao Shang, Zirui Liu, Qiwei Jiang, Zilin Ren, Ningyi Jin, Xiao Li, Zongzheng Zhao

**Affiliations:** ^1^Changchun Veterinary Research Institute, Chinese Academy of Agriculture Sciences, Changchun 130122, China; ^2^College of Veterinary Medicine, Hebei Agricultural University, Baoding 071000, China; ^3^Institute of Special Animal and Plant Sciences, Chinese Academy of Agricultural Sciences, Changchun 130122, China; ^4^College of Animal Science and Technology, Tarim University, Alar 843300, China; ^5^College of Animal Science and Technology, Guangxi University, Nanning 530004, China; ^6^College of Veterinary Medicine, Jilin Agricultural University, Changchun 130118, China

## Abstract

The H5N6 avian influenza virus (AIV) is constantly undergoing recombination and evolution with other subtypes of AIV, resulting in various types of recombinant H5N6 viruses. However, the risk to human public health of different recombinant types of H5N6 viruses remains unclear. Recently, two types of different recombinant H5N6 viruses were isolated from chickens. One of the viruses possessed six internal genes originating from H9N2, named A/Chicken/Hubei/112/2020 (H5N6) (abbreviated 112); the other virus possessed PB2, PB1, PA, and NP originating from H5N1, while the M and NS genes were derived from H9N2, named A/Chicken/Hubei/125/2020 (H5N6) (abbreviated 125). Here, we investigated the receptor binding properties, pathogenicity, and transmissibility of the two H5N6 AIVs. The results showed that 112 and 125 could bind *α*-2,3-linked sialic acid receptor (avian-like receptor) and *α*-2,6-linked sialic acid receptor (human-like receptor). However, 125 and 112 showed different pathogenicity in mice. Mice infected with 125 lost only a slight body weight and all survived, while mice infected with 112 lost weight rapidly and all died within a week of infection. Furthermore, in the transmission experiment, 125 could only transmit through direct contact, while 112 could transmit not only by direct contact but also by aerosol. The above results indicated that 112 exhibited enhanced pathogenicity and transmissibility compared to 125, suggesting that the H5N6 virus, whose internal genes were derived from H9N2, could pose a greater threat to human health. Therefore, continuous monitoring of different recombinant H5N6 viruses in poultry should be carried out to prevent their transmission to humans.

## 1. Introduction

The continuous evolution of the influenza A virus (IAV) poses a huge challenge to human and animal health. Wild waterfowl are natural hosts of IAVs, and migratory birds are drivers of IAV transmission. They play an important role in the evolution and spread of recombinant IAVs [1]. The avian influenza virus (AIV) can occasionally cross the species barrier and be transmitted from birds to mammals. However, it currently does not have the ability to be transmitted from human to human. Genetic recombination is an important mechanism for the production of novel influenza viruses with unique phenotypes. This process involves the exchange of genetic material between different strains of the virus, leading to the emergence of new variants [2].

In recent years, incidents of human infection with H5N6 have been reported from time to time, posing a significant risk to public health. Previous studies have shown that H5N6 originates from recombination between the H5 subtype and the H6N6 virus, and the H5N6 genes come mainly from two lineages; one is from H5 AIVs, such as H5N1, H5N2, H5N8, etc.; another is from low-pathogenic AIVs (LPAIVs), including H9N2, H6N6, etc. [3]. The six internal genes of some H5N6 viruses are all derived from H9N2 viruses. Some H5N6 viruses have internal genes derived from other LPAIVs, while others have internal genes that originate from the H5 subtype [4].

The H9N2 virus has spread and posed significant challenges to the poultry industry and human health [5]. As the most prevalent subtype of AIV in poultry, the H9N2 virus can infect poultry and mammals, recombine with other subtypes of AIV, and participate directly or indirectly in the emergence of the next influenza pandemic. This virus has also caused significant economic losses to the poultry industry. This subtype of the virus is also responsible for generating internal protein-encoding gene segments, which play a key role in the emergence of new strains [6]. On the one hand, H9N2 can cause damage to poultry through direct infection, coinfection, and immunosuppression. On the other hand, H9N2 has been found to share some or even all of its internal genes with emerging strains of H5N1, H7N9, H10N8, and H5N6 [6, 7]. In poultry, H9N2 can serve as a genetic incubator for new recombinant AIV, which has contributed to the emergence and evolution of new human infections with AIV [8]. It is important to note that the H9N2 virus is becoming increasingly adaptable to chickens and mammals, including humans. This adaptability increases the likelihood of a pandemic in the population [6].

Since their first recognition, high-pathogenic H5N1 AIV (HPAI H5N1) has become endemic in poultry in Southeast Asia and has spread to more countries [9]. H5N6 was first documented in Laos in 2013 and then subsequently in Vietnam and China, resulting in multiple poultry H5N6 outbreaks [4, 10]. Before human infection with H5N1, it was widely believed that AIVs that could bind *α*-2,6-sialic acid receptors could infect humans, but those that can only bind *α*-2,3-sialic acid could not infect humans [11, 12]. H5N6 has been shown to bind *α*-2,3-sialic acid and *α*-2,6-sialic acid receptors and could also attach to the human tracheal epithelium and alveolar tissue [7]. Therefore, H5N6 has a higher probability of infecting mammals or humans and carries higher public health and safety risks compared to H5N1, as evidenced by recent scientific studies and experiments.

The transmission and prevalence of H9N2, H5N1, and H5N6 in poultry have provided the possibility of the generation of different types of recombinant H5N6. Here, two types of recombinant H5N6 viruses were isolated from poultry, but the potential risk to human health of the two H5N6 viruses remains unclear. In this study, we analyzed the genetic evolution and homology of the genes of the two viruses, and receptor-binding properties, pathogenicity, and transmissibility of the two H5N6 viruses were also evaluated. The results add to our understanding of the pathogenicity and transmissibility of H5N6 viruses and will contribute to the prevention of AIVs.

## 2. Materials and Methods

### 2.1. Moral Statement

All animal studies were conducted in strict accordance with the guidelines of animal welfare of the World Organization for Animal Health (WOAH). Experimental protocols involving animals were approved by the Animal Care and Use Committee of Changchun Veterinary Research Institute (approval number: SCXK 20200001). All experiments with H5N6 viruses were performed in a biosecurity level 3 laboratory approved by Changchun Veterinary Research Institute of the Chinese Academy of Agricultural Sciences.

### 2.2. Virus

Two H5N6 viruses were isolated from poultry. The virus isolates were A/Chicken/Hubei/112/2020 (H5N6) (referred to as 112) and A/Chicken/Hubei/125/2020 (H5N6) (referred to as 125). The 2009 pandemic H1N1 influenza virus, A/Mexico/4486/2009 (H1N1), referred to as pdm09-H1N1 was a human-infected strain; H9N2 AIV was isolated from chicken by our laboratory. The viruses were grown in 9-day-old pathogen-free embryonated chicken eggs for 72 h at 37°C, collected allantoic fluid, and stored at −80°C.

### 2.3. Phylogenetic and Homology Analyses

Viral RNA was extracted from the allantoic fluid using the Tengen kit and reverse transcribed to complementary DNA (cDNA) using primer Uni12 (5′-AGCRAAAGCAGG-3′). Polymerase chain reaction (PCR) products of all gene fragments (PB2, PB1, PA, hemagglutination [HA], NP, neuraminidase [NA], M, and NS) of the H5N6 viruses were amplified using specific viral primers using SANGER sequencing technology. PCR products were purified by Comate Bioscience Company Limited, and sequence data were analyzed using the SEQMAN program (DNASTAR, Madison, Wisconsin, USA). All reference sequences used in this study were obtained from the National Centre for Biotechnology Information (NCBI) GenBank database. Phylogenetic analyses were performed by distance-based neighbor-joining method using MEGA11 software (DNA Star, Inc.).

### 2.4. Receptor-Binding Specificity Assay

The receptor-binding specificities of the H5N6 viruses were determined by HA assays. In each well of a microtiter plate, 50 μL of phosphate-buffered saline (PBS) was added. In the first row, taking 50 μL of the test sample and serially diluting by transferring 50 μL from the first well to successive wells. After that, 50 μL of red blood cell (RBC) suspension was added to each well on the plate. Both cells and virus controls were kept on the same plate. The plate was incubated at room temperature (RT) as described previously [13]. For the HA assay, sialic acid residues were enzymatically removed from chicken RBCs (cRBCs) by incubating them with 50 mU of *Vibrio cholerae* neuraminidase (VCNA, Roche, San Francisco, CA, USA) at 37°C for 1 h. Afterward, the cells were resialylated using either *α*-2,6-N-sialyltransferase or *α*-2,3-N-sialyltransferase (Sigma-Aldrich, St. Louis, MO, USA) at 37°C for 4 h. The sample was then washed two times with PBS, centrifuged at 1500 rpm for 5 min each time, adjusted to a final working concentration (1%) with PBS, and stored at 4°C. Viruses were serially diluted two-fold with 50 μL of PBS and mixed with 50 μL of a 1% RBC suspension in a 96-well plate. HA titers were determined after 1 h at 4°C.

### 2.5. Mouse Experiments

Groups of five 6-week-old female BALB/c mice (Merial Vital Laboratory Animal Technology Company) were anesthetized with ether and intranasally inoculated with 50 μL of the H5N6 viruses at 10^5.0^ EID_50_. The weight loss and survival rates of mice in these groups were monitored daily for 14 days. The percentages of body weight change for each group were calculated by comparing the group's average weight with their initial average weight. Mice that lost >30% of their original body weight were humanely euthanized.

For the assessment of viral growth and pathological changes in the lungs of the infected mice, 12 mice per group were anesthetized with ether and intranasally inoculated with the H5N6 viruses at 10^5.0^ EID_50_, while another three mice were intranasally inoculated with PBS served as the control. Three mice were euthanized at 1, 3, 5, and 7 days postinfection (dpi). The lungs of these mice were removed to determine the viral titers. Briefly, the lung tissues were weighed, and 0.1 g of each tissue was placed into 1 mL of PBS containing 100 U/mL penicillin, making 10% weight/volume lung homogenates. The tissue samples were homogenized by Tissue Lyser (QIAGEN, Germany) and centrifuged at 12,000 rpm. The supernatants were then collected and inoculated into 9-day-old pathogen-free embryonated chicken eggs. After 72 h of incubation at 37°C, the hemagglutinin activity was tested, and the EID_50_ was determined by the Reed method. At 3 dpi, the lungs of the infected mice were fixed in formalin, embedded in paraffin, and stained with hematoxylin and eosin (H&E) for pathological examination.

### 2.6. Guinea Pig Experiments

Hartley strain female guinea pigs (Merial Vital Laboratory Animal Technology Company) weighing 300–350 g were used for the transmission study. All guinea pigs were divided into three groups, with three in each group, namely the infection group, the direct contact group, and the aerosol group. Isoflurane anesthesia was used, and 200 μL of 10^6.0^ EID_50_ virus liquid was inoculated intranasally with three healthy guinea pigs in the infected group by pipette and was housed in a cage with an isolator.

One day after being infected, three healthy guinea pigs were housed in the guinea pig cage of the infected group, and each infected guinea pig was paired with one healthy guinea pig separately for a direct contact transmission study. Every infected guinea pig was housed at intervals with one healthy guinea pig through two iron nets 5 cm apart for aerosol transmission study. To monitor virus shedding, nasal washes were collected from all animals at 2, 4, 6, and 8 dpi and titrated in 9-day-old pathogen-free embryonated chicken eggs. After 72 h incubation at 37°C, the EID_50_ was determined by the Reed and Muench method.

All test animals were kept in the same environment as the control animals, with sufficient water and food, and bedding was changed regularly. At the end of the test, all test animals were anesthetized with isoflurane and sacrificed according to animal welfare standards.

### 2.7. Statistics Analysis

Statistically significant differences were determined using one-way analysis of variance (ANOVA) with GraphPad Prism software (San Diego, CA, USA). All the assays were run in triplicate and were representative of at least three separate experiments. The error bars represent the standard deviation.

## 3. Result

### 3.1. Phylogenetic Analysis

**Figure 1:**
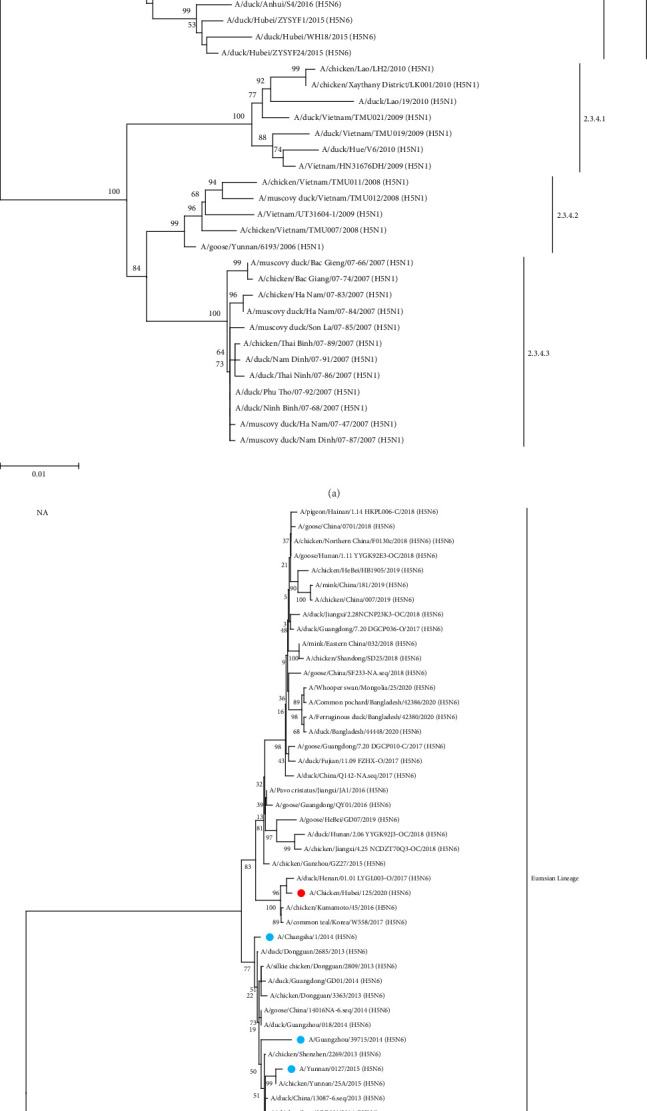
Phylogenetic analysis of the HA and NA genes. The phylogenetic trees of the HA (A) and NA (B) genes were constructed using the distance-based neighbor-joining method in the MEGA11 software. The reliability of the trees was assessed by bootstrap analysis. Horizontal distances are proportional to genetic distances. 112 and 125 are marked with red dots. The H5N6 viruses of human infections are marked with blue dots. HA, hemagglutination; NA, neuraminidase.

To study the genetic relationship between the two H5N6 viruses, we downloaded the AIV gene sequence from the NCBI database, constructed a genetic evolution tree based on the nucleotide sequences of HA and NA, and phylogenetic analysis was performed using MEGA11 software. The results showed that the HA genes of 112 and 125 were clustered in the branch of the H5 subtype 2.3.4.4h, which was in the same evolutionary branches as the H5N6 viruses isolated from humans (Figure 1A). The NA genes of 112 and 125 were from the Eurasian lineage branch and were in the same evolutionary branch as the NA of H5N6 infected with humans. The NA of 125 was close to the NA of A/duck/Henan/01.01 LYGL003-O/2017(H5N6), and the NA of 112 was close to the NA of A/chicken/Guangdong/GD1602/2016(H5N6) and A/chicken/Anhui/MZ33/2016(H5N6) (Figure 1B). In both H5N6 virus isolates, the alteration of HA protein at G228S implied a preference for avian-like (*α*-2,3-linked salivary acid receptors) receptor binding. Simultaneously, the existence of T160A mutations indicated that the virus may have increased affinity for human-like (*α*-2,6-linked salivary acid receptors) receptor binding. In addition, in both strains, the stem domain of NA protein in the NA stem region was found to have 11 amino acids missing, signifying that the virus exhibits some adaptability and pathogenicity in mammals. In addition, E627K mutations were observed in the PB2 protein of 112 and not in 125, which was associated with polymerase activity and increased mammalian fitness. These results suggest that the HA and NA of 112 and 125 may have a common ancestor with those of H5N6 infected with humans.

### 3.2. Homology Analysis

Homology analysis was performed between the nucleotide sequences of two H5N6 virus strains and the influenza virus gene sequences downloaded from the NCBI database. The HA genes of 112 and 125 had 97.77% and 97.94% similarity to the HA genes of A/Changsha/1/2014(H5N6), respectively, and the NA gene had 97.68% and 97.25% similarity to the NA gene of A/Changsha/1/2014(H5N6), respectively (Table 1). The M gene of 112 and 125 had 99.02% and 99.04% similarity to A/chicken/Dongguan/1674/2014(H9N2), respectively, and the NS gene had 99.16% and 99.40% similarity to A/chicken/Hubei/S0301/2016(H9N2), respectively (Table 1). These results suggest that both the M and NS genes of 112 and 125 were derived from H9N2.

The 112 polymerase genes PB2, PB1, PA, and NP had 99.06%, 98.36%, 98.59%, and 98.92% similarity to A/chicken/Zhejiang/SIC30/2014(H9N2), A/chicken/Qingdao/2044/2013(H9N2), A/chicken/Zhejiang/C38/2013(H9N2), A/chicken/China/E620/2014(H9N2), respectively. The 125 polymerase genes PB2, PB1, PA, and NP had 96.25%, 99.02%, 98.17%, and 99.04% similarity to A/duck/Shanghai/20180606/2017(H5N1), A/duck/Shanghai/ZXC01/2017(H5N1), A/duck/Vietnam/LBM639/2014(H5N1), A/duck/Shanghai/20180603/2017(H5N1), respectively (Table 2).

**Figure 2:**
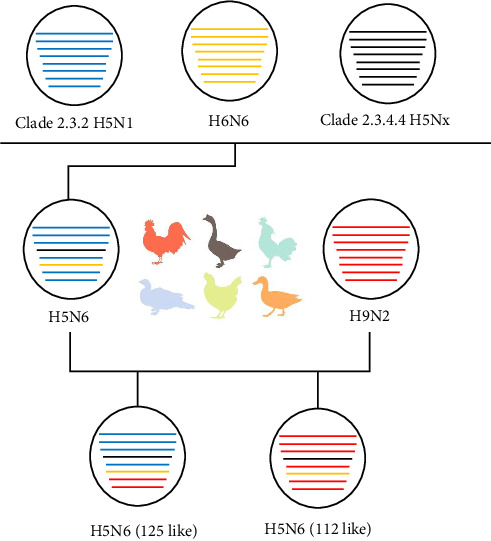
Genesis of H5N6 AIV. Virus particles are shown as colored ovals containing horizontal bars that represent the eight gene segments (from top to bottom: PB2, PB1, PA, HA, NP, NA, M, and NS). To illustrate the history of reassortant events, segments in descendant viruses are colored according to their corresponding source viruses. AIV, avian influenza virus; HA, hemagglutination; NA, neuraminidase.

We conducted homology analyses of the viruses to determine the recombinant evolutionary process of the two strains of H5N6 viruses sampled in this study, which may reveal fragments of the complex evolutionary origins of the H5N6 viruses (Figure 2). Specifically, present-day H5N6 viruses originated as recombinations of clade 2.3.2 H5N1, H6N6, and clade 2.3.4.4 H5Nx. These viruses continue to spread in poultry and recombine with H9N2 to form the two recombinant types of H5N6 viruses observed in this study. The internal genes of 112 were all derived from H9N2, while the M and NS genes of 125 were also derived from H9N2, and the polymerase genes PB2, PB1, PA, and NP of 125 were derived from H5N1.

In summary, the HA and NA genes of the 112 and 125 viruses show high homology to the H5N6 viruses that infect humans and a variety of internal gene sources. The main difference in the composition of the 112 and 125 genes was that the polymerase genes of the two viruses were derived from H9N2 and H5N1, respectively.

### 3.3. Receptor Binding Assay

**Figure 3:**
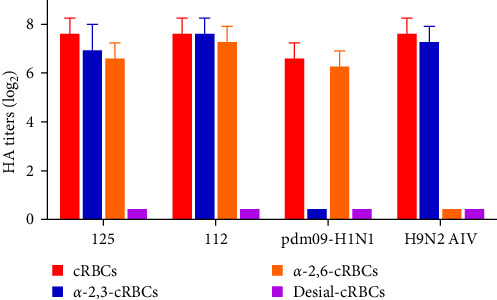
Agglutination activities of the 125 and 112 viruses in various cRBCs. Four types of cRBCs were used: (a) cRBCs. (b) *α*-2,3 cRBCs (treated with VCNA and resialylated with *α*-2,3 glycans). (c) *α*-2,6 cRBCs (treated with VCNA and resialylated with *α*-2,6 glycans). (d) Desial-cRBCs (treated with VCNA). The HA titers demonstrated the agglutination activities of the 125 and 112 in the four types of cRBCs. The reported values are presented as the means and standard deviations of three independent experiments. AIV, avian influenza virus; cRBCs, chicken red blood cells; HA, hemagglutination; VCNA, *Vibrio cholerae* neuraminidase.

To investigate the receptor-binding properties of 125 and 112, the receptor-binding specificity of 125 and 112 for *α*-2,3-linked sialic acid receptor and *α*-2,6-linked sialic acid receptor using HA assays. We measured the receptor binding specificity of the two viruses as previously described [14]. cRBCs contain *α*-2,3-linked sialic acid receptor and *α*-2,6-linked sialic acid receptor, while cRBCs treated with VCNA contain no receptors (Desial-cRBCs), and *α*-2,6-cRBCs or *α*-2,3-cRBCs contained either *α*-2,6-linked sialic acid (human-like receptor) or *α*-2,3-linked sialic acid (avian-like receptor) receptors. The results showed that cRBCs, *α*-2,3-cRBCs, and *α*-2,6-cRBCs could be agglutinated by 125 and 112, but Desiial-cRBCs could not be agglutinated by 125 and 112 (Figure 3), suggesting that 125 and 112 could bind to both *α*-2,3-linked sialic acid receptor and *α*-2,6-linked sialic acid receptor.

### 3.4. Pathogenicity of the H5N6 Viruses in Mice

**Figure 4:**
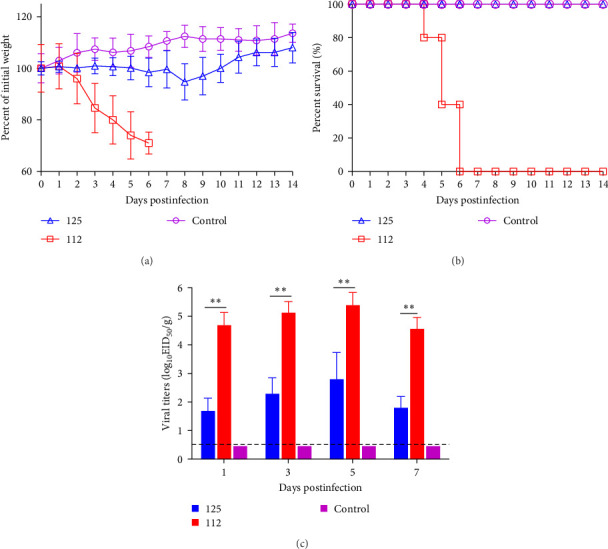
Pathogenicity of 125 and 112 in mice. Five mice per group were intranasally inoculated with virus 105.0 EID50. Viral titers in the homogenized tissue supernatants were determined by the Reed-Muench method. The results were presented as the mean ± SD, all data were analyzed using the one-way ANOVA method, and the comparisons between two groups were made using the SNK method. (A) Mice were weighed daily for 14 days. (B) Mouse survival was calculated by observing deaths. (C) Virus titers in the lungs of mice. *⁣*^*∗*^*p* < 0.05, *⁣*^*∗∗*^*p* < 0.01 (125 vs. 112).

To assess the pathogenicity of 125 and 112 in mammals, five 6-week-old female BALB/c mice were infected with 125 and 112 at 10^5.0^ EID_50_, respectively. The changes in body weight and survival were monitored for 14 days. Mice inoculated with 125 lost weight slowly, dropping ~5% of their initial body weight at 8 dpi and then gradually recovering. However, mice infected with 112 lost weight rapidly, and more than 30% of their initial body weight was lost (mice that lost more than 30% of their initial body weight during the experiment were euthanized), whereas the weight of the control mice continued to increase (Figure 4A). The survival rate was also analyzed. Mice infected with 112 had a mortality rate of 20% (1/5) at 4 dpi, 60% (3/5) at 5 dpi, and 100% (5/5) at 6 dpi, while mice infected with 125 and control mice did not show any deaths within 14 days (Figure 4B), and the above data indicate that 112 is more pathogenic than125 in mice.

The viral titers in the lungs of mice infected with 125 and 112 were also compared. The viral titers in the lungs of mice infected with 125 were 10^1.7^ EID_50_/g, 10^2.3^ EID_50_/g, 10^2.8^ EID_50_/g, and 10^1.8^ EID_50_/g at 1, 3, 5, and 7 dpi, respectively, whereas the virus titers of 112 were 10^4.7^ EID_50_/g, 10^5.1^ EID_50_/g, 10^5.4^ EID_50_/g and 10^4.6^ EID_50_/g at 1, 3, 5, and 7 dpi. The viral titers of 112 were significantly higher than those of 125 (*p*  < 0.01) (Figure 4C). The above results suggest that 112 has a better replication capacity in the lungs of mice than 125, which may be a reason why 112 is more pathogenic than 125.

**Figure 5:**
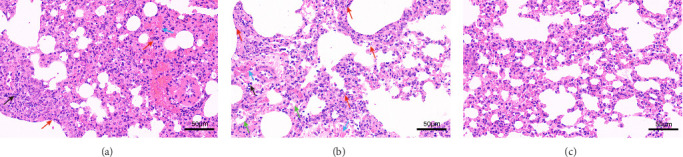
Histopathological analysis. The lungs of infected mice were fixed with formalin, embedded in paraffin, and stained with hematoxylin and eosin. (A) Lung tissue sections of mice infected with 125. (B) Lung tissue sections of mice infected with 112. (C) Lung tissue sections from control mice. The images were acquired using ×200 magnification. Alveolar atrophy and collapse (green arrows); infiltration of inflammatory cells (black arrows); cell necrosis, cell degeneration, and nuclear fragmentation (red arrows); erythrocyte exudation (blue arrows).

In addition, we also conducted a histopathological analysis of the lungs of mice. The lung tissue of mice infected with 125 and 112 both displayed cell necrosis, cell degeneration, nuclear fragmentation (as shown by the red arrows), infiltration of inflammatory cells (as shown by the black arrows), and erythrocyte exudation (as shown by the blue arrows) (Figure 5A,B). However, the lung tissue of 112 infected mice also displayed atrophy and collapse of alveoli (as shown by the green arrows) (Figure 5B), whereas the lungs of control mice showed no obvious pathological changes (Figure 5C). Histopathological analysis showed that mice infected with 112 exhibited more severe histopathological changes than mice infected with 125.

### 3.5. Evaluation of Transmission Capacity of H5N6 Among Guinea Pigs

**Figure 6:**
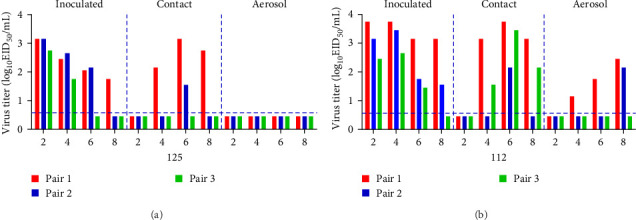
Horizontal transmissions of 125 (A) and 112 (B) viruses in guinea pigs. Three guinea pigs per group were inoculated with the specified viruses. The next day, the three inoculated guinea pigs were individually paired by cohousing with a direct contact guinea pig; also, an aerosol contact guinea pig was housed in a wire frame cage adjacent to that of the infected guinea pig. The distance between the cages of the infected and aerosol-contact guinea pigs was 5 cm apart. Nasal washes were collected from all animals every other day for virus-shedding assays. The dashed line indicates the lower limit of virus detection.

Guinea pigs were used to determine the transmissibility of 125 and 112. Nasal washes were collected from guinea pigs in all infected, direct-contact, and aerosol groups, and viral titers were determined in 9-day-old chick embryos. 125 was detectable in all infected groups of guinea pigs (3/3). 125 was also detected in two of the direct-contact guinea pigs, one at 4, 6, and 8 dpi and the other at 6 dpi, whereas the virus could not be detected in guinea pigs in the aerosol group (Figure 6A). 112 could be detected in all infected groups and in direct-contact guinea pigs. 112 could be detected in two guinea pigs in the direct-contact group at 4 and 8 dpi and in three guinea pigs direct-contact group at 6 dpi. 112 was detected in two guinea pigs in the aerosol group. 112 could be detected in one guinea pig of the aerosol group at 4 and 6 dpi and in two guinea pigs of the aerosol group at 8 dpi (Figure 6B). These results show that 125 could be transmitted by direct contact in mammals. 112 could be transmitted by both direct contact and aerosol. 112 has an enhanced transmissibility compared to 125.

## 4. Discussion

The H5N6 virus has attracted significant attention due to its ability to infect humans and cause fatalities. According to epidemiological investigations, almost all patients infected with H5N6 have a history of contact with poultry [15]. Although H5N6 spreads in poultry, the possibility of human infection is unavoidable [16, 17]. Previous studies have found that multiple types of recombinant H5N6 viruses have been detected in human infections in China, indicating a high degree of genetic compatibility between H5N6 and other AIVs [15, 17]. Genetic recombination of AIVs is very common in wild birds, but stable genomic evolution occurs in poultry [18, 19]. Due to the migration routes, habitat geography, and ecological distribution of birds, as well as complex herd immunity, progressively AIV carried by birds accumulate amino acid substitution, while stable host transformations such as poultry, horses, pigs, and humans lead to several poorly characterized mutations that separate single clonal influenza virus strains from the AIV gene pool of large wild birds [20]. Notably, the combination of large poultry populations allows natural selection to effectively drive rapid antigenic and genetic changes within a single subtype, while recombination with the AIV gene pool carried in wild birds contributes to the generation of a new genome pool [18]. Poultry plays an important role in the evolution of new recombinant AIVs, and stable genetic evolution is a characteristic of AIVs for adapting to mammals [18, 19]. The prevalence of the H5N6 virus in wild birds enables the virus to recombine with LPAIV in wild birds. Furthermore, the migration of wild birds leads to the potential for interspecific transmission and adaptation, which poses a significant risk to human health [21, 22]. Some H5N6 viruses have been reported to bind *α*-2,6 and *α*-2,3-linked salivary acid receptors [23, 24], thus breaking the species barrier to infecting humans [25]. IAV enters host cells through specific recognition of the sialic acid receptor. *α*-2,6-linked sialic acid receptors are abundant in the human upper respiratory tract [26, 27]. However, most AIVs have a preference for *α*-2,3-linked sialic acid receptors, which are abundant in the intestinal mucosa of avian species [11, 28–30]. The difference in receptor binding preferences is considered one of the main reasons why AIVs are rarely transmitted to humans, and human influenza viruses isolated from humans could not replicate well in avian hosts [31]. Recombination of H5 AIV with other subtypes of AIV may promote its transmission to mammals, but further adaptations may be required for successful mammalian adaptation [32, 33]. H5N6 viruses have been shown to bind to *α*-2,6 sialic acid receptors [34], and H5N6 could also be transmitted through direct contact among mammals [13, 35, 36].

In this study, we found that the H5N6 viruses 112 and 125 were able to bind the *α*-2,6-sialic acid receptor. 125 and 112 could also be transmitted by direct contact among mammals. Furthermore, 112 could be transmitted by aerosol transmission among mammals. The results suggest that H5N6 in poultry continues to evolve, which poses a serious risk to public health and safety.

H9N2 is prevalent in poultry and is constantly mutating and evolving, and its adaptability to both poultry and mammals is also constantly strengthening [7]. H9N2 can not only directly infect animals and humans but also provide some or even all of the internal genes necessary for other AIV subtypes to generate new recombinant viruses, such as H5N1, H7N9, H10N8, and H5N6 [37]. The internal genes of H9N2 provide the potential for novel recombinant viruses to infect humans [7, 38–41]. In this study, all internal genes of 112 were derived from H9N2, while the polymerase genes of 125 were derived from H5N1. 112 exhibits enhanced pathogenicity and transmissibility compared to 125. This indicates that H5N6, containing H9N2 internal genes, may have better adaptation to mammals.

H9N2 has caused huge economic losses to the poultry industry despite long-term vaccination programs. Since 2014, the combined epidemic of H9N2 and H5N6 has led to a sharp increase in the chance that it may infect mammals, which poses a huge threat to public health safety. The currently circulating H9N2 virus has a so-called pandemic potential, and its key feature is the change of receptor-binding properties of HA protein from avian-like to human-like, which is considered a prerequisite for efficient human-to-human transmission [42–44]. However, the prevalence of H9N2 AIV has increased year by year. Furthermore, the unorganized transportation of poultry carrying H9N2 throughout the country and the recombination between different subtypes of H9 and other subtypes have made the circulation of H9 in China very complex [8]. In addition, H9N2 has been reported in other mammalian species, such as pigs, dogs, horses, and mink. Individuals infected with H9N2 exhibit mild flu-like symptoms, including respiratory symptoms, cough, fever, nasal discharge, sore throat, and headache. As a result, mild infections are often overlooked, giving H9N2 a good opportunity and time to obtain mutations, thereby increasing its adaptation among humans [45]. In fact, traces of H9N2 have been found in several reports of avian influenza infection in humans. In the 1997 H5N1 epidemic in Hong Kong, it was found that the H9N2 virus contributed six internal genes [46]. In 2012, a strain of HPAI H5N1, belonging to clade 2.3.2.1a and isolated in Bhutan, with the H9-like polymerase basic protein 1 (M1) gene, replicates faster in vitro than its H5N1 parental genotype and spreads more efficiently in chicken models [47]. Since 2013, H9N2 has contributed six internal genes for the H7N9 and H10N8 viruses that appear in Chinese human infection [48, 49]. In Egypt, the cocirculation of H5N1 and H9N2 in birds provides an opportunity for recombination between the two viruses [50]. Repeated human infections with H9N2 will inevitably increase the likelihood that it will acquire adaptive mutations, resulting in pandemics.

In conclusion, H5N6 viruses with recombinant internal genes derived from H9N2 have shown increased pathogenicity and transmissibility. It is of great importance to pay attention to the recombination of internal H9 AIV genes with genes from other AIV subtypes. Therefore, the continuous evolution of the H5N6 virus is a major concern for both the poultry industry and public health. In particular, the number of human infections with H5N6 has continued to increase in recent years; the reason for this is that H5N6 undergoes genetic recombination and adaptive mutations in mammals, suggesting that the ongoing evolution of H5N6 can increase the risk of human infections.

## Figures and Tables

**Table 1:** The HA, NA, M, and NS of strains 112 and 125 were analyzed for sequence homology with H5N6, a virus capable of infecting humans.

The strains were used in this study		A/chicken/Hubei/112/2020(H5N6)	A/chicken/Hubei/125/2020(H5N6)
Strain (accession number)	Gene	Similarity (%)	
A/Changsha/1/2014(H5N6)(KR063687.1)	HA	97.77	97.94

A/Changsha/1/2014(H5N6)(KR063689.1)	NA	97.68	97.25

A/chicken/Dongguan/1674/2014(H9N2) (KP416449.1)	M	99.02	99.04

A/chicken/Hubei/S0301/2016(H9N2) (MN647226.1)	NS	99.16	99.40

Abbreviations: HA, hemagglutination; NA, neuraminidase.

**Table 2:** Homology analysis of PB2, PB1, PA, and NP genes of 112 and 125.

The strains were used in this study	Gene	Similarity (%)	Strains	Accession number
A/chicken/Hubei/112/2020(H5N6)	PB2	99.06	A/chicken/Zhejiang/SIC30/2014(H9N2)	KX598537.1
	PB1	98.36	A/chicken/Qingdao/2044/2013(H9N2)	KP415161.1
	PA	98.59	A/chicken/Zhejiang/C38/2013(H9N2)	KU042228.1
	NP	98.92	A/chicken/China/E620/2014(H9N2)	MN100490.1

A/chicken/Hubei/125/2020(H5N6)	PB2	96.25	A/duck/Shanghai/20180606/2017(H5N1)	MH341596.1
	PB1	99.02	A/duck/Shanghai/ZXC01/2017(H5N1)	MH341604.1
	PA	98.17	A/duck/Vietnam/LBM639/2014(H5N1)	AB979510.1
	NP	99.04	A/duck/Shanghai/20180603/2017(H5N1)	MH341594.1

## Data Availability

The authors confirm that the data supporting the findings of this study are available within the article.
